# Alcohol consumption and carotid artery structure in Korean adults aged 50 years and older

**DOI:** 10.1186/1471-2458-9-358

**Published:** 2009-09-23

**Authors:** Young-Hoon Lee, Min-Ho Shin, Sun-Seog Kweon, Sung-Woo Choi, Hye-Yeon Kim, So-Yeon Ryu, Bok-Hee Kim, Jung-Ae Rhee, Jin-Su Choi

**Affiliations:** 1 Department of Preventive Medicine, Seonam University College of Medicine, 720, Kwangchi-dong, Namwon, Jeollabukdo 590-711, South Korea; 2 Department of Preventive Medicine, Chonnam National University Medical School, 5, Hak-1-dong, Dong-gu, Gwangju 501-746, South Korea; 3 Jeonnam Regional Cancer Center, Chonnam National University Hwasun Hospital, 160, Ilsim-ri, Hwasun-eup, Hwasun-gun, Jeollanamdo 519-809, South Korea; 4 Department of Preventive Medicine, College of Medicine, Chosun University, 375, Seosuk-dong, Dong-gu, Gwangju 501-759, South Korea; 5 Department of Food & Nutrition, College of Natural Sciences, Chosun University, 375, Seosuk-dong, Dong-gu, Gwangju 501-759, South Korea

## Abstract

**Background:**

Epidemiologic studies of the association between alcohol consumption and carotid artery structure have reported conflicting results. We investigated the association between alcohol consumption and carotid atherosclerosis by evaluating the effects of alcohol intake on carotid artery enlargement.

**Methods:**

The study population consisted of 4302 community-dwelling Koreans (1577 men and 2725 women) aged 50 years and over. All the subjects had participated in the baseline survey of the Dong-gu Study conducted between 2007 and 2008. Daily alcohol consumption was determined by the number and frequency of alcoholic beverages consumed. We measured common carotid artery intima-media thickness (CCA-IMT), common carotid and bulb IMT (CB-IMT), carotid plaques, and the diameter of the common carotid artery (CCA-diameter) using high-resolution B-mode ultrasonography. We used analysis of covariance and multiple logistic regressions to determine the relationship between alcohol consumption and carotid artery parameters.

**Results:**

CCA-IMT and CB-IMT were negatively correlated with alcohol consumption after controlling for cardiovascular risk factors in men (*p *for linear trend = 0.009 and = 0.038, respectively). The multivariate-adjusted odds ratio (OR) for carotid plaques was significantly higher in men who consumed >40.0 g/d (OR = 1.81, 95% CI = 1.13-2.91), although a significant positive correlation was observed between alcohol consumption and carotid plaques (*p *for linear trend = 0.027). Neither carotid IMT nor carotid plaques were correlated with alcohol intake in women. Alcohol intake was positively correlated with CCA-diameter adjusted for carotid IMT and plaques in the multivariate-adjusted model in both sexes (*p *for linear trend <0.001 for men and 0.020 for women).

**Conclusion:**

The results of our study indicate that alcohol consumption is inversely related to carotid IMT and positively related to carotid plaques in men, but not women. However, alcohol intake is positively associated with CCA-diameter in both men and women. Additional large population-based prospective studies are needed to confirm the effects of alcohol consumption on carotid artery structure.

## Background

Cumulative epidemiologic evidence indicates a J-shaped or a U-shaped association between alcohol consumption and cardiovascular disease morbidity and mortality [1-4]. Light to moderate drinking (1 or 2 drinks daily for men and 1 drink daily for women) is associated with cardiovascular protective effects, whereas excessive alcohol intake (>2 drinks daily) results in poor health outcomes [3,4]. High-resolution B-mode ultrasonography can detect changes in the arterial wall structure, including intima-media thickness (IMT), atherosclerotic plaque, and arterial diameter. Carotid IMT is a surrogate marker for subclinical atherosclerosis and a strong predictor of future cardiovascular events such as myocardial infarction and stroke [5-8]. Because carotid IMT can be measured safely, simply, and noninvasively using high-resolution B-mode ultrasonography, the technique is being increasingly used as an endpoint in epidemiological and intervention studies. Several studies have investigated the effects of alcohol consumption on carotid artery structure, but the results have been conflicting. Some studies have found an association between alcohol consumption and carotid IMT and carotid plaques [9-13], whereas others reported no relationship between alcohol intake and carotid atherosclerosis [14-16]. Moreover, few studies have investigated the relationship between alcohol intake and carotid artery enlargement [16-19] and thus the effects of alcohol consumption on the structure of the carotid arterial wall remain unclear.

The objective of this study was to investigate the relationship between alcohol consumption and carotid atherosclerosis. We evaluated the effects of alcohol intake on carotid arterial diameter in a cross-sectional study of subjects aged 50 years and older.

## Methods

### Subjects

The study population consisted of 4302 community-dwelling Korean men and women aged 50 years and over. The subjects were participants in the baseline survey of the Dong-gu Study conducted between 2007 and 2008. The Dong-gu Study is an ongoing prospective study that was designed to investigate the prevalence, incidence, and risk factors for chronic disease in the urban elderly. We used the national resident registration to identify potential participants. In total, 17670 eligible subjects (7905 men and 9765 women) aged 50 years and over, who resided in five town in the Dong-gu district of the Gwangju Metropolitan City of South Korea, were invited by telephone to participate. Of these, 4302 subjects (1577 men and 2725 women; response rate, 24.3%) underwent clinical examinations following interviews. The response rate of women (27.9%) was significantly higher than that of men (19.9%), but no significant mean age difference existed between the men and women who participated and those who did not. Two hundred and ten subjects were excluded from the study because of missing information about alcohol consumption or poor ultrasonographic images of their carotid artery parameters. In total, 4092 subjects were included in the study (1492 men and 2600 women). The present study was conducted in accordance with the Declaration of Helsinki guidelines, and informed consent for the procedure was obtained from each subject. The study protocol was approved by the institutional review board of Chonnam National University Hospital.

### Alcohol consumption

Alcohol intake was assessed using a structured interview, including four questions. The following two questions were used to determine the current drinking status of the study population: "Prior to the date of this study, have you ever drunk alcoholic beverages?" and "Do you presently drink alcoholic beverages (in the 12 months prior to this interview)?" Participants who answered "no" to both questions were classified as never-drinkers (lifetime abstainers). Participants who answered "yes" to the first question and "no" to the second question were classified as former drinkers. Current drinkers were defined as participants who answered "yes" to both questions. Current drinkers additionally answered two related questions: "On a day when you do drink alcohol, how many drinks do you usually have?" and "How often do you have a drink containing alcohol, per month?" The amount of ethanol consumed per day was calculated from the average number of alcoholic beverages consumed. Because 'soju' is the most widely consumed traditional beverage in South Korea, the average amount of alcohol for each beverage type was converted into the corresponding equivalents of soju (1 unit of soju = 10 g of ethanol) using an alcoholic beverage conversion table. We divided participants into six categories on the basis of daily alcohol consumption. The categories for men were never, former, 0.1-10.0 g/d, 10.1-20.0 g/d, 20.1-40.0 g/d, and >40.0 g/d and for women were never, former, 0.1-5.0 g/d, 5.1-10.0 g/d, 10.1-20.0 g/d, and >20.0 g/d.

### Carotid ultrasonography

Two trained technicians who were blind to the subject groups evaluated the carotid artery structure in all participants using high-resolution B-mode ultrasound (SONOACE 9900, Medison, Seoul, Korea) equipped with a 7.5 MHz linear array transducer. Images of the common carotid artery (CCA), carotid bulb, and internal carotid artery were used to evaluate IMT, plaque, and arterial diameter. A single trained reader analyzed the frozen images using SigmaScan Pro Version 5.0.0 (SPSS Inc., Chicago, IL, USA) according to a standardized protocol. The ultrasound parameters used in this study were CCA-IMT, CB-IMT, carotid plaques, and diameter of the common carotid artery (CCA-diameter). IMT was determined as the distance from the media-adventitia interface to the intima-lumen interface on the far wall in a region free of plaques. Between the carotid bulb origin and a point 10 mm proximal to the common carotid artery on the longitudinal view (10 mm in length), we performed multiple (two to five) measurements to determine the maximal IMT of the CCA. The maximal value of two to five measurements was determined as 'the maximal IMT value of the left/right CCA.' We also performed multiple (two to five) IMT measurements between the origin of the carotid bulb and the origin of the internal carotid artery to determine the maximal IMT of the carotid bulb. The maximal value of two to five measurements was determined as 'the maximal IMT value of the left/right carotid bulb.' Finally, 'CCA-IMT,' defined as the average of the maximal values of both CCA and 'CB-IMT,' defined as the average of the maximal values of the four arterial segments, including the CCA and bulb of the left and right carotid artery, was used for analysis. Higher CCA-IMT was defined as CCA-IMT ≥ 1.0 mm [20]. The reader also assessed the presence of carotid plaques--defined as focal structures that encroached into the lumen by at least 100% of the surrounding IMT value. The presence of carotid plaques was determined from the scans of the carotid artery segments included in the study (common, bulb, and internal carotid artery). The presence of carotid plaques was recorded if at least one lesion was detected in any segment. CCA diameter was defined as the distance between the near wall periadventitia-to-adventitia interface and the far wall adventitia-to-periadventitia interface at a point 10 mm proximal to the beginning of the dilation of the carotid bulb (bulb origin) in a longitudinal view. We evaluated consecutive images of the carotid artery during a 10-s phase (video clip) and stored images of the minimum common carotid artery diameter, representing end-diastolic phase. Two ultrasonography technicians conducted repeated ultrasound examinations in 189 subjects to ensure measurement reproducibility. The correlation coefficients for between and within examiner variability were 0.86 and 0.90, respectively for CCA-IMT and 0.87 and 0.95, respectively, for CCA-diameter. The kappa coefficients were 0.76 for between-examiner agreement and 0.85 for within-examiner agreement.

### Covariates

We collected information on each subject's medical history and lifestyle characteristics using standardized questionnaires. Smoking status classifications were current smokers, former smokers, and never-smokers. Physical exercise was assessed by asking the frequency of recreational activity and exercise over 30 min during a week. Physical exercise was categorized as none (0-1 time per week), irregular exercise (2-4 times per week), and regular exercise (5 or more times per week). All participants underwent a standardized physical examination performed by experienced research staff. Anthropometric measurements were conducted in light clothing and without shoes. Height was measured to the nearest 0.1 cm, and weight was measured with a standard scale in the upright position to the nearest 0.1 kg. Body mass index (BMI) was calculated as weight (kg) divided by height squared (m^2^). Waist circumference was measured to the nearest 0.1 cm at the midpoint between the lower border of the rib cage and the upper hip bone (iliac crest) during expiration. Blood pressure was measured after at least 5 min rest in the sitting position with an appropriate size cuff on the right upper arm using a standard mercury sphygmomanometer (Baumanometer; WA Baum Co., Inc., Copiague, NY, USA). Three readings of systolic and diastolic blood pressure were recorded at 1-min intervals and the average was used in the analysis. Hypertension was defined as systolic blood pressure ≥140 mm Hg or diastolic blood pressure ≥90 mm Hg, or use of antihypertensive drugs. Blood samples taken from the antecubital vein were collected from each subject in the morning after a 12-h overnight fast. Serum was separated from the samples within 30 min and stored at -70°C until use for analysis. Serum total cholesterol, high-density lipoprotein (HDL) cholesterol, triglyceride, and fasting blood glucose levels were analyzed using the enzymatic method. Enzyme activities for gamma-glutamyl transpeptidase (GGT) and aspartate aminotransferase (AST) were measured using commercial reagent kits (Daiichi Pure Chemicals, Tokyo, Japan). All samples were measured using an automatic analyzer (model 7600 chemical analyzer; Hitachi Ltd., Tokyo, Japan). Diabetes was defined as a fasting blood glucose level ≥126 mg/dl or use of medication for diabetes. Hypercholesterolemia was defined as a total cholesterol level ≥240 mg/dl or use of lipid-lowering drugs.

### Statistical analysis

All analyses in this study were performed separately for men and women because of the differences in the amount of alcohol consumed and sex-specific differences in alcohol metabolism. Demographic and clinical characteristics of the study population were expressed as the mean ± standard deviation or as a ratio based on the alcohol consumption categories. Carotid artery measurements of CCA-IMT, CB-IMT, and CCA-diameter were expressed as the mean ± standard error of the mean. We used analyses of variance (ANOVAs) to determine statistical differences in the continuous variables and chi-square tests to determine significant differences in the discrete variables. The relationship between alcohol consumption and the carotid artery parameters was tested using an analysis of covariance (CCA-IMT, CB-IMT, CCA-diameter) or a multiple logistic regression analysis (higher CCA-IMT, carotid plaques). After excluding former drinkers, alcohol consumption categories were treated as a continuous variable to test for a linear trend in the relationship between alcohol intake and carotid artery parameters. All statistical analyses were performed using SPSS software version 15.0.

## Results

### Characteristics of the study population

Of the 4092 subjects analyzed, 1492 (36.5%) were men and 2600 (63.5%) were women. The mean age of the men in this study was 66.7 ± 7.6 years (range 50-88 years), and the mean age of the women was 64.8 ± 8.1 years (range 50-90 years). Within the study population, 69.4% of men and 32.9% of women were current alcohol drinkers, whereas 16.6% of men and 60.7% of women did not drink. The average amount of alcohol consumed in grams per day was 12.9 ± 23.1 g/d for men and 1.3 ± 4.8 g/d for women. Demographic and clinical characteristics of the study population and the categories of daily alcohol consumption are shown in Table 1 for men and Table 2 for women.

**Table 1:** Characteristics According to Alcohol Consumption in Men (n = 1492)

	**Categories of Alcohol Consumption**						
		
	**Never** **(n = 248)**	**Former** **(n = 208)**	**0.1-10.0 g/d** **(n = 638)**	**10.1-20.0 g/d** **(n = 152)**	**20.1-40.0 g/d** **(n = 114)**	**> 40.0 g/d** **(n = 132)**	p*
Age, y	68.8 ± 7.7	68.8 ± 7.8	66.1 ± 7.3	65.6 ± 7.6	65.4 ± 6.8	64.2 ± 7.7	< 0.001
BMI, kg/m^2^	23.3 ± 3.1	23.5 ± 2.9	23.8 ± 2.6	24.2 ± 2.6	24.3 ± 2.8	23.9 ± 2.9	0.003
WC, cm	86.0 ± 8.7	86.8 ± 8.3	86.7 ± 7.2	88.4 ± 7.2	89.5 ± 7.6	87.9 ± 8.0	< 0.001
SBP, mm Hg	122.2 ± 15.5	121.5 ± 15.9	122.0 ± 16.1	123.8 ± 13.1	124.9 ± 16.1	126.8 ± 16.5	0.012
DBP, mm Hg	71.7 ± 8.4	71.3 ± 9.4	72.5 ± 9.9	73.5 ± 8.2	76.5 ± 10.0	75.7 ± 11.0	< 0.001
FBG, mg/dL	107.6 ± 24.8	111.3 ± 27.6	109.4 ± 24.9	112.1 ± 21.4	111.7 ± 21.6	117.6 ± 30.8	0.007
Total cholesterol, mg/dL	179.1 ± 37.0	173.4 ± 35.0	182.6 ± 35.5	180.3 ± 33.5	192.5 ± 40.2	180.1 ± 38.8	< 0.001
HDL cholesterol, mg/dL	47.5 ± 11.3	45.2 ± 10.5	49.0 ± 11.6	50.5 ± 11.8	53.7 ± 14.7	54.6 ± 16.0	< 0.001
Triglycerides, mg/dL	126.5 ± 81.1	130.4 ± 74.0	130.9 ± 87.6	137.6 ± 87.8	179.5 ± 259.4	194.9 ± 175.3	< 0.001
AST, U/L	23.8 ± 10.9	23.3 ± 12.0	22.4 ± 7.1	26.0 ± 15.6	26.8 ± 16.4	39.1 ± 46.8	< 0.001
GGT, U/L	27.3 ± 23.3	29.4 ± 23.8	36.5 ± 56.7	47.4 ± 68.9	67.0 ± 112.4	153.3 ± 291.0	< 0.001
Hypertension, %	39.5	52.4	38.9	46.1	48.2	53.0	0.001
Diabetes, %	23.8	27.4	20.1	23.7	21.1	28.0	0.176
Hypercholesterolemia, %	12.9	13.0	10.7	11.8	14.0	15.2	0.683
Current smoking, %	17.3	20.2	20.1	27.6	28.1	41.7	< 0.001
Regular exercise, %	29.4	32.2	38.2	29.6	32.5	19.7	0.001

Data are means ± standard deviations.

BMI, body mass index; WC, waist circumference; SBP, systolic blood pressure; DBP, diastolic blood pressure; FBG, fasting blood glucose; HDL, high-density lipoprotein; AST, aspartate aminotransferase; GGT, gamma-glutamyl transferase.

*p for difference was obtained by analysis of variance for continuous variables and chi-square test for categorical variables, respectively.

**Table 2:** Characteristics According to Alcohol Consumption in Women (n = 2600)

	**Categories of Alcohol Consumption**						
		
	**Never** **(n = 1577)**	**Former** **(n = 167)**	**0.1-5.0 g/d** **(n = 715)**	**5.1-10.0 g/d** **(n = 60)**	**10.1-20.0 g/d** **(n = 56)**	**>20.0 g/d** **(n = 25)**	p*
Age, y	65.8 ± 8.0	67.3 ± 7.8	62.5 ± 7.6	61.6 ± 7.5	62.3 ± 9.0	59.9 ± 7.9	< 0.001
BMI, kg/m^2^	24.5 ± 3.1	24.4 ± 3.0	24.5 ± 2.8	24.3 ± 2.7	24.3 ± 2.9	25.0 ± 2.6	0.908
WC, cm	91.9 ± 8.3	91.0 ± 8.4	91.0 ± 7.8	90.1 ± 6.9	91.0 ± 7.7	92.3 ± 7.7	0.065
SBP, mm Hg	120.7 ± 15.6	122.1 ± 15.9	120.0 ± 17.0	117.8 ± 13.8	118.9 ± 16.1	122.8 ± 17.0	0.354
DBP, mm Hg	71.5 ± 9.0	71.8 ± 9.3	71.6 ± 9.8	70.7 ± 10.7	71.3 ± 10.0	75.3 ± 10.4	0.449
FBG, mg/dL	104.5 ± 22.7	106.7 ± 25.4	106.3 ± 24.5	107.4 ± 25.1	100.7 ± 10.5	112.3 ± 30.3	0.118
Total cholesterol, mg/dL	201.7 ± 38.3	201.9 ± 40.5	202.6 ± 36.3	202.2 ± 37.4	195.7 ± 29.2	217.4 ± 60.7	0.310
HDL cholesterol, mg/dL	52.2 ± 11.7	50.3 ± 11.1	53.6 ± 12.6	54.9 ± 13.5	56.7 ± 11.0	59.3 ± 15.7	< 0.001
Triglycerides, mg/dL	140.6 ± 80.2	154.5 ± 101.9	143.9 ± 91.0	117.4 ± 67.0	121.6 ± 65.6	244.9 ± 406.6	< 0.001
AST, U/L	22.3 ± 9.1	24.4 ± 18.4	22.0 ± 7.1	22.7 ± 7.3	22.1 ± 5.7	24.0 ± 18.6	0.090
GGT, U/L	20.8 ± 29.5	23.2 ± 22.6	22.7 ± 21.4	32.9 ± 68.8	26.2 ± 24.3	33.9 ± 44.0	0.004
Hypertension, %	41.7	48.5	39.6	28.3	39.3	24.0	0.037
Diabetes, %	15.3	19.8	14.5	15.0	10.7	16.0	0.578
Hypercholesterolemia, %	24.5	26.3	23.5	26.7	12.5	36.0	0.225
Current smoking, %	1.5	2.4	2.5	6.7	3.6	4.0	0.060
Regular exercise, %	23.2	24.6	25.2	18.3	28.6	28.0	0.697

Data are means ± standard deviations.

BMI, body mass index; WC, waist circumference; SBP, systolic blood pressure; DBP, diastolic blood pressure; FBG, fasting blood glucose; HDL, high-density lipoprotein; AST, aspartate aminotransferase; GGT, gamma-glutamyl transferase.

*p for difference was obtained by analysis of variance for continuous variables and chi-square test for categorical variables, respectively.

### Alcohol consumption and carotid IMT parameters

The relationship between alcohol consumption and the carotid IMT parameters of CCA-IMT, CB-IMT, and higher CCA-IMT are shown in Table 3 for men and Table 4 for women. CCA-IMT was significantly greater in men (0.779 ± 0.156 mm) than in women (0.731 ± 0.142 mm), and CB-IMT was also significantly greater in men (0.847 ± 0.139 mm) than in women (0.789 ± 0.132 mm). In men, CCA-IMT had a significant negative correlation with alcohol consumption in an age-adjusted and multivariate-adjusted model (*p *for linear trend = 0.028 and <0.001, respectively) (Table 3). In addition, CB-IMT was inversely related to alcohol consumption in the age-adjusted and multivariate-adjusted analysis in men (*p *for linear trend = 0.018 and 0.001, respectively). Alcohol intake was inversely related to CCA-IMT and CB-IMT in models controlling for HDL cholesterol (*p *for linear trend = 0.009 and 0.038, respectively). When subjects were divided into two groups according to CCA-IMT (higher CCA-IMT ≥ 1.0 mm and lower CCA-IMT <1.0 mm), we observed a significant decrease in the prevalence of higher CCA-IMT with alcohol consumption in men (*p *< 0.001). The age-adjusted and multivariate-adjusted odds ratio (OR) of higher CCA-IMT was significantly lowered by alcohol intake in men (*p *for linear trend = 0.007 and 0.001, respectively). The inverse relationship between alcohol consumption and higher CCA-IMT was also observed in the analysis adjusted for HDL cholesterol (*p *for linear trend = 0.004). Multivariate analysis using higher CCA-IMT confirmed an independent adverse relationship between alcohol consumption and carotid IMT. However, in women, alcohol consumption was not associated with CCA-IMT or CB-IMT in age-adjusted, multivariate-adjusted, or HDL cholesterol-adjusted analyses. In addition, no association was found between alcohol intake and higher CCA-IMT in women (Table 4).

**Table 3:** Relation between Alcohol Consumption and Carotid IMT Parameters in Men

	**Categories of Alcohol Consumption**						
		
	**Never** **(n = 248)**	***Former*** **(*n *= 208)**	**0.1-10.0 g/d** **(n = 638)**	**10.1-20.0 g/d** **(n = 152)**	**20.1-40.0** **g/d (n = 114)**	**> 40.0 g/d** **(n = 132)**	***p*¶**
CCA-IMT, mm							
Age-adjusted	0.805 ± 0.009	0.778 ± 0.010*	0.775 ± 0.006**	0.773 ± 0.012*	0.770 ± 0.014*	0.769 ± 0.013*	0.028
Multivariate-adjusted†	0.814 ± 0.009	0.782 ± 0.010*	0.776 ± 0.006**	0.767 ± 0.012**	0.756 ± 0.013**	0.758 ± 0.013**	< 0.001
Multivariate-adjusted+HDL‡	0.811 ± 0.009	0.778 ± 0.010*	0.775 ± 0.006**	0.769 ± 0.012**	0.762 ± 0.013**	0.769 ± 0.013**	0.009
CB-IMT, mm							
Age-adjusted	0.868 ± 0.008	*0.848 ± 0.009*	0.843 ± 0.005**	0.842 ± 0.011*	0.850 ± 0.012	0.827 ± 0.011**	0.018
Multivariate-adjusted†	0.874 ± 0.008	*0.852 ± 0.009*	0.843 ± 0.005**	0.839 ± 0.010**	0.841 ± 0.012*	0.821 ± 0.011**	0.001
Multivariate-adjusted+HDL‡	0.871 ± 0.008	*0.848 ± 0.009*	0.842 ± 0.005**	0.841 ± 0.010*	0.847 ± 0.012	0.832 ± 0.012**	0.038
Higher CCA-IMT§							
Prevalence, %	27.8	24.5	19.1	17.1	16.7	12.1	< 0.001
Age-adjusted	1	0.83 (*0.54-1.28*)	0.72 (0.51-1.03)	0.64 (0.38-1.08)	0.64 (0.36-1.14)	0.47 (0.26-0.85)*	0.007
Multivariate-adjusted†	1	0.78 (*0.50-1.22*)	0.68 (0.47-0.98)*	0.56 (0.32-0.95)*	0.50 (0.28-0.92)*	0.38 (0.20-0.71)*	0.001
Multivariate-adjusted+HDL‡	1	0.78 (*0.49-1.22*)	0.68 (0.47-0.99)*	0.57 (0.33-0.98)*	0.53 (0.29-0.98)*	0.41 (0.21-0.78)*	0.004

Data are means ± standard error of mean or odds ratios (95% confidence interval).

*p < 0.05, **p < 0.01: compared with never-drinkers.

†Analysis adjusted for age, body mass index, waist circumference, smoking status, exercise, systolic blood pressure, diastolic blood pressure, total cholesterol, triglycerides (log transformed), fasting glucose, use of medication for hypertension, use of medication for diabetes, and use of medication for hyperlipidemia.

‡Analysis additionally adjusted for HDL cholesterol.

§CCA-IMT ≥ 1.0 mm.

¶*p *for linear trend was obtained by analysis of covariance or logistic regression using the categories of alcohol consumption as a continuous variable (excluding former drinkers).

**Table 4:** Relation between Alcohol Consumption and Carotid IMT Parameters in Women

	**Categories of Alcohol Consumption**						
		
	**Never** **(n = 1577)**	***Former*** **(*n *= 167)**	**0.1-5.0 g/d** **(n = 715)**	**5.1-10.0 g/d** **(n = 60)**	**10.1-20.0 g/d** **(n = 56)**	**>20.0 g/d** **(n = 25)**	***p*¶**
CCA-IMT, mm							
Age-adjusted	0.731 ± 0.003	*0.735 ± 0.010*	0.730 ± 0.005	0.737 ± 0.017	0.729 ± 0.017	0.721 ± 0.026	0.691
Multivariate-adjusted†	0.732 ± 0.003	*0.736 ± 0.010*	0.729 ± 0.005	0.735 ± 0.016	0.730 ± 0.017	0.707 ± 0.025	0.364
Multivariate-adjusted+HDL‡	0.731 ± 0.003	*0.734 ± 0.010*	0.730 ± 0.005	0.735 ± 0.016	0.735 ± 0.017	0.715 ± 0.025	0.608
CB-IMT, mm							
Age-adjusted	0.789 ± 0.003	*0.787 ± 0.009*	0.788 ± 0.005	0.792 ± 0.016	0.778 ± 0.016	0.763 ± 0.024	0.225
Multivariate-adjusted†	0.790 ± 0.003	*0.787 ± 0.009*	0.787 ± 0.004	0.790 ± 0.015	0.779 ± 0.016	0.751 ± 0.024	0.087
Multivariate-adjusted+HDL‡	0.790 ± 0.003	*0.785 ± 0.009*	0.788 ± 0.004	0.790 ± 0.015	0.783 ± 0.016	0.759 ± 0.024	0.189
Higher CCA-IMT§							
Prevalence, %	13.3	17.4	10.1	11.7	10.7	8.0	0.037
Age-adjusted	1	1.25 (*0.81-1.93*)	0.95 (0.71-1.28)	1.22 (0.54-2.79)	0.98 (0.40-2.37)	0.89 (0.20-3.95)	0.867
Multivariate-adjusted†	1	1.28 (*0.82-2.00*)	0.94 (0.69-1.27)	1.34 (0.59-3.08)	1.04 (0.42-2.54)	0.78 (0.17-3.64)	0.846
Multivariate-adjusted+HDL‡	1	1.20 (*0.77-1.89*)	0.99 (0.73-1.34)	1.39 (0.60-3.20)	1.21 (0.49-2.99)	0.99 (0.22-4.53)	0.743

Data are means ± standard error of mean or odds ratios (95% confidence interval).

*p < 0.05, **p < 0.01: compared with never-drinkers.

† Analysis adjusted for age, body mass index, waist circumference, smoking status, exercise, systolic blood pressure, diastolic blood pressure, total cholesterol, triglycerides (log transformed), fasting glucose, use of medication for hypertension, use of medication for diabetes, and use of medication for hyperlipidemia.

‡Analysis additionally adjusted for HDL cholesterol.

§CCA-IMT ≥1.0 mm.

¶*p *for linear trend was obtained by analysis of covariance or logistic regression using the categories of alcohol consumption as a continuous variable (excluding former drinkers).

### Alcohol consumption and carotid plaques

The prevalence of carotid plaques was positively correlated with daily alcohol intake in men, although the correlation was not statistically significant (Table 5). A significant positive correlation was observed between alcohol consumption and carotid plaques in the age-adjusted, but not the multivariate-adjusted, analysis in men (*p *for linear trend = 0.003 and 0.080, respectively). When we added HDL cholesterol or the CCA-IMT in the multivariate model, the relationship between alcohol intake and carotid plaques remained significant (*p *for linear trend = 0.049 and 0.027, respectively). Multivariate plus HDL cholesterol-adjusted and multivariate plus CCA-IMT-adjusted ORs for carotid plaques were significantly higher only in men who consumed >40.0 g/d (OR = 1.72, 95% CI = 1.06-2.79 and OR = 1.81, 95% CI = 1.13-2.91, respectively). However, no significant relationship between alcohol consumption and carotid plaques was observed in women (Table 6).

**Table 5:** Relation between Alcohol Consumption and Carotid Plaques, CCA-diameter in Men

	**Categories of Alcohol Consumption**						
		
	**Never** **(n = 248)**	***Former*** **(*n *= 208)**	**0.1-10.0 g/d** **(n = 638)**	**10.1-20.0** **g/d (n = 152)**	**20.1-40.0 g/d** **(n = 114)**	**> 40.0 g/d** **(n = 132)**	***p *∫**
Carotid plaques							
Prevalence, %	46.4	55.8	42.5	46.7	45.6	56.1	0.125
Age-adjusted	1	1.48 (1.01-2.17)*	0.99 (0.73-1.35)	1.22 (0.81-1.86)	1.18 (0.75-1.87)	1.97 (1.27-3.07)**	0.003
Multivariate-adjusted†	1	*1.46 (0.98-2.16)*	1.02 (0.74-1.40)	1.17 (0.76-1.80)	1.08 (0.67-1.75)	1.64 (1.03-2.62)*	0.080
Multivariate-adjusted+HDL‡	1	*1.45 (0.98-2.15)*	1.03 (0.75-1.41)	1.19 (0.77-1.84)	1.12 (0.69-1.81)	1.72 (1.06-2.79)*	0.049
Multivariate-adjusted+CCA-IMT§	1	1.54 (1.03-2.29)*	1.09 (0.79-1.51)	1.25 (0.81-1.94)	1.20 (0.74-1.95)	1.81 (1.13-2.91)*	0.027
CCA-diameter, mm							
Age-adjusted	7.797 ± 0.053	7.997 ± 0.058**	7.925 ± 0.033*	8.113 ± 0.067**	8.166 ± 0.077**	8.241 ± 0.072**	<0.001
Multivariate-adjusted†	7.883 ± 0.047	*8.001 ± 0.051*	7.961 ± 0.029	8.013 ± 0.059	8.061 ± 0.068*	8.092 ± 0.066**	0.005
Multivariate-adjusted+HDL‡	7.881 ± 0.047	*7.999 ± 0.051*	7.960 ± 0.029	8.014 ± 0.059	8.065 ± 0.068*	8.099 ± 0.066**	0.006
Multivariate-adjusted+CCA-IMT, plaques¶	7.838 ± 0.045	7.990 ± 0.048*	7.968 ± 0.027*	8.029 ± 0.056**	8.095 ± 0.065**	8.108 ± 0.062**	<0.001

Data are means ± standard error of mean or odds ratios (95% confidence interval).

*p < 0.05, **p < 0.01: compared with never-drinkers.

† Analysis adjusted for age, body mass index, waist circumference, smoking status, exercise, systolic blood pressure, diastolic blood pressure, total cholesterol, triglycerides (log transformed), fasting glucose, use of medication for hypertension, use of medication for diabetes, and use of medication for hyperlipidemia.

‡ Analysis additionally adjusted for HDL cholesterol.

§Analysis additionally adjusted for CCA-IMT.

¶Analysis additionally adjusted for CCA-IMT and carotid plaques.

∫*p *for linear trend was obtained by analysis of covariance or logistic regression using the categories of alcohol consumption as a continuous variable (excluding former drinkers).

**Table 6:** Relation between Alcohol Consumption and Carotid Plaques, CCA-diameter in Women

	**Categories of Alcohol Consumption**						
		
	**Never** **(n = 1577)**	***Former*** **(*n *= 167)**	**0.1-5.0 g/d** **(n = 715)**	**5.1-10.0 g/d** **(n = 60)**	**10.1-20.0** **g/d (n = 56)**	**>20.0 g/d** **(n = 25)**	***p *∫**
Carotid plaques							
Prevalence, %	46.4	55.8	42.5	46.7	45.6	56.1	0.222
Age-adjusted	1	*1.27 (0.89-1.81)*	1.14 (0.92-1.41)	0.65 (0.31-1.36)	1.18 (0.62-2.23)	1.34 (0.52-3.46)	0.365
Multivariate-adjusted†	1	*1.23 (0.85-1.77)*	1.09 (0.87-1.36)	0.70 (0.33-1.47)	1.18 (0.62-2.27)	1.19 (0.45-3.18)	0.590
Multivariate-adjusted+HDL‡	1	*1.20 (0.83-1.74)*	1.11 (0.89-1.38)	0.71 (0.34-1.48)	1.24 (0.65-2.38)	1.29 (0.48-3.44)	0.434
Multivariate-adjusted+CCA-IMT§	1	*1.23 (0.85-1.78)*	1.09 (0.88-1.37)	0.69 (0.33-1.45)	1.19 (0.62-2.28)	1.25 (0.47-3.36)	0.566
CCA-diameter, mm							
Age-adjusted	7.492 ± 0.017	*7.587 ± 0.052*	7.530 ± 0.026	7.529 ± 0.087	7.544 ± 0.090	7.788 ± 0.135*	0.035
Multivariate-adjusted†	7.497 ± 0.016	*7.577 ± 0.048*	7.523 ± 0.023	7.547 ± 0.080	7.531 ± 0.082	7.749 ± 0.123*	0.051
Multivariate-adjusted+HDL‡	7.496 ± 0.016	*7.572 ± 0.048*	7.525 ± 0.023	7.547 ± 0.080	7.539 ± 0.082	7.764 ± 0.123*	0.036
Multivariate-adjusted+CCA-IMT, plaques¶	7.496 ± 0.015	*7.569 ± 0.046*	7.526 ± 0.022	7.544 ± 0.076	7.532 ± 0.078	7.784 ± 0.118*	0.020

Data are means ± standard error of mean or odds ratios (95% confidence interval).

*p < 0.05, **p < 0.01: compared with never-drinkers.

†Analysis adjusted for age, body mass index, waist circumference, smoking status, exercise, systolic blood pressure, diastolic blood pressure, total cholesterol, triglycerides (log transformed), fasting glucose, use of medication for hypertension, use of medication for diabetes, and use of medication for hyperlipidemia.

‡Analysis additionally adjusted for HDL cholesterol.

§Analysis additionally adjusted for CCA-IMT.

¶Analysis additionally adjusted for CCA-IMT and carotid plaques.

∫*p *for linear trend was obtained by analysis of covariance or logistic regression using the categories of alcohol consumption as a continuous variable (excluding former drinkers).

### Alcohol consumption and CCA-diameter

The CCA-diameter was significantly greater in men (7.979 ± 0.854 mm) than in women (7.514 ± 0.733 mm). Alcohol consumption was positively correlated with CCA-diameter in the age-adjusted model for both sexes (*p *for linear trend <0.001 for men and 0.035 for women) (Tables 5, 6). In the multivariate-adjusted model, the association remained significant in men but was only borderline significant in women (*p *for linear trend = 0.005 for men and 0.051 for women). When we added HDL cholesterol to the multivariate model, the relationship between alcohol intake and CCA-diameter remained significant in both men and women (*p *for linear trend = 0.006 and 0.036, respectively). Furthermore, when CCA-IMT and carotid plaques were added into the multivariate model, the association between alcohol consumption and CCA-diameter also remained statistically significant for men and women (*p *for linear trend <0.001 for men and 0.020 for women). In men, the CCA-diameter in all the alcohol consumption categories, even former drinkers, was significantly larger than that of nondrinkers. In contrast, a significant increase in the CCA-diameter was found only in women consuming >20 g/d compared to nondrinkers (Table 6).

## Discussion

The results of our study suggest that after controlling for multiple risk factors, high alcohol consumption is associated with a decrease in CCA-IMT and an increase in the occurrence of carotid plaques in men. Neither CCA-IMT nor carotid plaques were correlated with alcohol intake in women. However, we observed that CCA diameter was positively correlated with alcohol intake independent of conventional cardiovascular risk factors in both men and women.

The relationship between alcohol consumption and carotid atherosclerosis is unclear. Several population-based epidemiologic studies have investigated the effects of alcohol consumption on carotid atherosclerosis, but their findings were not in agreement. The Cardiovascular Health Study of 5888 adults aged 65 years and older [11] found an inverse relationship between alcohol consumption and carotid atherosclerosis in subjects who consumed 1-6 drinks per week (equalling <15 g/d), whereas the correlation was positive for subjects who consumed 14 or more drinks per week (equalling >30 g/d). The Study of Health in Pomerania [13] reported a J-shaped relationship between alcohol consumption and carotid IMT in men but not in women. The Portugal study [12], found a lower incidence of carotid plaque in moderate drinkers (101-300 ml/wk), suggesting that moderate alcohol consumption has an anti-atherogenic effect. The results from cross-sectional and longitudinal data in the Bruneck Study [9,10] showed a J-shaped relationship between regular alcohol intake and carotid atherosclerosis as defined by plaque and vessel stenosis. In contrast, the Cardiovascular Risk in Young Finns Study [21] found that alcohol consumption was positively associated with carotid IMT in young adults aged 24-39 years after controlling for age, sex, and cardiovascular risk factors. This finding suggests that alcohol consumption has a pro-atherogenic effect. However, the ARIC study [14] did not observe a cross-sectional association between current alcohol consumption and carotid IMT. The NHLBI Family Heart Study [15] and the Three-City Study [16] did not find a significant relationship between alcohol intake and carotid IMT. The results of our study differed from those of previous studies. We did not find the J-shaped association between alcohol consumption and carotid atherosclerosis described by several epidemiologic studies. Instead, we observed a linear decrease in carotid IMT and a linear increase in carotid plaques with alcohol intake in men, but not women. We believe that this discrepancy may be explained, at least in part, by differences in histological characteristics and the stage of carotid atherosclerosis under study. Several epidemiologic studies have shown that the natural history, pattern of risk factors, and the prediction of cardiovascular events are different for carotid IMT and carotid plaques, even though they share many common atherosclerotic risk factors [22,23]. In our study, alcohol consumption was inversely related to CCA-IMT in men, suggesting that increased alcohol consumption has a beneficial effect early in the atherosclerotic process. In contrast, alcohol consumption was positively correlated with the occurrence of carotid plaques. A higher incidence of carotid plaques was observed in heavy drinkers compared to nondrinkers, suggesting that increased alcohol consumption has a harmful effect on later stages of atherosclerosis. Recent studies have reported that carotid plaques are a better predictor of coronary artery disease, including myocardial infarction, than IMT [24,25]. Therefore, the effects of alcohol consumption on the progression of carotid IMT and carotid plaques should be carefully investigated in prospective epidemiologic studies.

Most previous studies have focused on the role of arterial luminal enlargement in compensating for thickening of the arterial wall. Some studies have suggested that the carotid artery may enlarge to compensate for arterial wall thickening and plaque formation to stabilize the shear stress caused at the interface between blood and the arterial endothelium occurring in the early stages of atherosclerosis [26-29].

Many studies have reported that arterial diameter is correlated with cardiovascular risk factors, such as systolic blood pressure, body mass index, smoking, alcohol consumption, blood lipids, and carotid artery IMT [17-19,30,31]. Furthermore, carotid enlargement has been considered as a surrogate end point of cardiovascular events. The Rotterdam Study reported a positive association of carotid lumen diameter with acute myocardial infarctions [32]. A larger lumen in diastole might reflect a lesser intrinsic vessel elasticity and thus a stiffer vessel, which might explain the positive association. The Three-City Study found that the increase in carotid distension was significantly predictive of CHD occurrence independently of age, sex, brachial and carotid PPs, heart rate, antihypertensive drugs, CCA-IMT, carotid plaques, and other major cardiovascular risk factors [33].

Most studies investigating the relationship between alcohol consumption and carotid atherosclerosis use carotid IMT and atherosclerotic plaques as surrogate markers of carotid atherosclerosis. Few studies have specifically examined the relationship between alcohol consumption and carotid artery diameter [16-19]. The Suita Study [18] and the EVA Study [17] reported a significant positive correlation between alcohol consumption and both outer and inner CCA diameters. In the Three-City Study [16], CCA-lumen diameter was positively correlated with alcohol consumption in both men and women after controlling for multiple risk factors, although no marked relationships of alcohol intake and carotid atherosclerosis were found. Moreover, when CCA-IMT and carotid plaques were added to the multivariate model, the relationship between alcohol consumption and CCA diameter remained statistically significant. Our results show that alcohol consumption is associated with enlarged arterial diameter even after multivariate adjustment for classical cardiovascular risk factors. The results of our study agree with those of several studies showing a positive association between alcohol intake and carotid diameter. We measured the outer diameter (distance between the two leading edges of the far wall and the near wall periadventitia-to-adventitia interfaces), but the results were similar for the inner diameter (distance between the two leading edges of the far wall and near wall intima-lumen interfaces) (data not shown). In our study, the relationship between alcohol consumption and CCA diameter was statistically significant after controlling for CCA-IMT and carotid plaques in the multivariate model. We confirmed that carotid artery enlargement in response to alcohol consumption is independent of carotid atherosclerosis, which was suggested by the Three-City Study. This finding indicates that alcohol can produce adaptive enlargement to protect the lumen from shear stress [16].

A potential weakness of epidemiologic studies on alcohol consumption is that self-reporting of alcohol use may not be accurate. A criticism of self-reporting is that it may lead to an underestimation of alcohol consumption, particularly by heavy drinkers. In this study, we validated self-reports of alcohol consumption by comparing alcohol intake with biochemical parameters such as AST, GGT, HDL cholesterol, and smoking habits, which are correlated with alcohol use [13]. Alcohol consumption was positively associated with AST, GGT, HDL cholesterol, and current smoking in men and positively associated with GGT and HDL cholesterol in women.

### Strengths and limitations

This study has several strengths. First, this study included a relatively large sample size. Second, we separated former drinkers who had stopped drinking for health or other reasons from nondrinkers. Former drinkers have different characteristics from nondrinkers, and analyzing their data separately provided more accurate results. Third, we measured the arterial IMT in both the common carotid and carotid bulb segments. Most previous studies have used the CCA-IMT as a marker for carotid atherosclerosis because it is highly reproducible and simple. However, we observed a similar correlation between alcohol intake and CCA-IMT and CB-IMT. Fourth, HDL cholesterol levels were controlled for in all analyses because it may indirectly affect alcohol use and carotid atherosclerosis. Similar results were observed in the adjusted multivariate model, suggesting direct effects of alcohol intake on carotid artery structure.

This study has certain limitations. First, an important limitation of our study is the low response rate (24.3%) of the residents of the study area, despite numerous contacts by telephone requesting participation. It is possible that many potential respondents overlooked the telephone invitations. The response rate of this study was considerably lower than that reported in other studies; it has been higher than 60% in some major studies. It is possible that a response rate of less than 30% does not reflect the status of the entire population. Second, we could not make causal inferences between alcohol consumption and modification of carotid arterial structure because of the cross-sectional design. Third, young adults aged 20-49 years old were not included in this study. Because the amount and frequency of alcohol consumption is generally lower for older adults than for younger adults in Korea, the strength of the relationship between alcohol consumption and carotid atherosclerosis might increase when young adults are included in the study population. Fourth, we determined the maximal IMT value at each arterial segment by a single maximal point selection, rather than the mean of multiple measurements. Single-point measurement of the IMT of each segment is more prone to measurement error than an average value of multiple measurements. Moreover, because we measured the IMT value manually, the reproducibility of our study was lower than that of other studies using automated edge-tracking software. Fifth, the absence of an association between alcohol intake and carotid atherosclerosis in women may be explained by the fact that women consume less alcohol than men. In addition, women drinking alcohol is not generally accepted in the Korean culture, and we speculate that underreporting would be higher among women than men. These measurement errors might have affected the relationship between alcohol consumption and CCA-IMT and carotid plaques in women.

## Conclusion

We conclude that self-reported alcohol consumption is inversely related to arterial wall thickening and positively correlated with the presence of carotid plaques in men but not in women. However, alcohol intake is positively correlated with CCA-diameter in both men and women. Additional large population-based prospective studies are needed to confirm the effects of alcohol consumption on carotid artery structure.

## Competing interests

The authors declare that they have no competing interests.

## Authors' contributions

YHL carried out physical examinations, analyzed the data, and drafted the manuscript. MHS and SSK coordinated the data collection and also performed physical examinations. SWC, HYK, SYR, and BHK conducted physical measurements and collected data. JAR and JSC participated in the design of the study, the data collection, and reviewed the manuscript. All authors read and approved the final manuscript to be published.

## Pre-publication history

The pre-publication history for this paper can be accessed here:


